# Risk for Misdiagnosing Chronic Traumatic Encephalopathy in Men With Anger Control Problems

**DOI:** 10.3389/fneur.2020.00739

**Published:** 2020-07-24

**Authors:** Grant L. Iverson, Andrew J. Gardner

**Affiliations:** ^1^Department of Physical Medicine and Rehabilitation, Harvard Medical School, Boston, MA, United States; ^2^Spaulding Rehabilitation Hospital and Spaulding Research Institute, Charlestown, MA, United States; ^3^MassGeneral Hospital for Children™ Sport Concussion Program, Boston, MA, United States; ^4^Home Base, A Red Sox Foundation and Massachusetts General Hospital Program, Charlestown, MA, United States; ^5^Hunter New England Local Health District, Sports Concussion Program, Newcastle, NSW, Australia; ^6^Priority Research Centre for Stroke and Brain Injury, School of Medicine and Public Health, University of Newcastle, Newcastle, NSW, Australia

**Keywords:** athletes, anger, depression, suicidal ideation, suicide, brain concussion, brain injuries, chronic traumatic encephalopathy

## Abstract

**Background:** There are no validated or agreed upon criteria for diagnosing chronic traumatic encephalopathy (CTE) in a living person. In recent years, it has been proposed that anger dyscontrol represents a behavioral clinical phenotype of CTE. This is the first study to examine the specificity of the diagnostic research criteria for traumatic encephalopathy syndrome (TES, the clinical condition proposed to be CTE) in men from the US general population who have anger dyscontrol problems. It was hypothesized that a substantial percentage of these men would meet the research criteria for TES.

**Methods:** Data from 4,139 men who participated in the National Comorbidity Survey Replication, an in-person survey that examined the prevalence and correlates of mental disorders in the United States, were included in this study. Men who were diagnosed with intermittent explosive disorder in the past year were the clinical sample of interest (*n* = 206; 5.0% of all men in the database), and the remaining men were used as a comparison sample. They were classified as meeting the research criteria for TES if they presented with the purported supportive clinical features of CTE (e.g., impulsivity/substance abuse, anxiety, apathy, suicidality, headache).

**Results:** In this sample of men from the general population with intermittent explosive disorder, 27.3% met a conservative definition of the proposed research criteria for CTE (i.e., traumatic encephalopathy syndrome). If one assumes the delayed-onset criterion is present, meaning that the men in the sample are compared to former athletes or military veterans presenting with mental health problems years after retirement, then 65.0% of this sample would meet the research criteria for TES.

**Conclusions:** These results have important implications. Using conservative criteria, at least one in four men from the general population, who have serious anger control problems, will meet the symptom criteria for TES. If one considers former athletes and military veterans with anger control problems who present many years after retirement and who experienced a documented decline in their mental health, nearly two-thirds will meet these research criteria. More research is needed to examine risks for misdiagnosing TES and to determine whether anger dyscontrol is a clinical phenotype of CTE.

## Introduction

There is tremendous interest in chronic traumatic encephalopathy (CTE). In the twentieth century, CTE was considered to be a neurological disorder affecting a subgroup of long-career boxers (1, 2), and the clinical features were usually described as reflecting rather obvious chronic brain damage and cognitive impairment (3, 4). Varying degrees of neurological hard signs, such as abnormal reflexes and hemiparesis, and extrapyramidal signs, such as slurred or dysarthric speech, gait abnormalities, and tremor, were described (2, 4–11). The extent to which CTE is static or progressive or whether its course reflects two or more different clinical conditions has never been clear (1, 2, 7, 10, 12–17), and many authors conceptualized CTE as a progressive parkinsonian-like neurological disorder, and others have not.

In its modern form, CTE is considered to be a postmortem neuropathological diagnosis (18, 19), with the defining pathological feature being the accumulation of hyperphosphorylated tau (p-tau), in a patchy distribution at the depths of the cortical sulci around small vessels (18, 19). This specific neuropathology has been identified after death in the brains of young athletes (20, 21), active NFL players (20, 21), former collegiate athletes from multiple sports (20), retired boxers (20), retired professional hockey players (20), retired NFL players (18, 20, 21), and military veterans (22). P-tau accumulates in the brain in normal aging and in numerous neurodegenerative diseases (23–28), but researchers have asserted that it does not accumulate in a *patchy distribution* in the depths of sulci in association with aging or other diseases (18, 19). However, this assertion remains in doubt because CTE neuropathology has been identified in some people from the general population with no known exposure to repetitive neurotrauma and in association with substance abuse, temporal lobe epilepsy, multiple system atrophy, amyotrophic lateral sclerosis, and other neurodegenerative diseases (29–36).

The extent to which the neuropathology of CTE causes specific clinical symptoms and problems is unclear (19, 37); there is no agreed-upon way to diagnose CTE in a living person, and there is major interest in developing and validating clinical diagnostic criteria. At present, validated diagnostic criteria do not exist, although several sets have been proposed (13, 38–40). Preliminary proposed research criteria for “traumatic encephalopathy syndrome (TES)” (39) include three core features of CTE: (i) “cognitive,” (ii) “behavioral” (i.e., anger dyscontrol), and (iii) “mood” (i.e., depression or hopelessness). These core features are used to define diagnostic “subtypes” or “variants” according to the research criteria. In addition to a subtype, two supportive features must be present [i.e., impulsivity, anxiety, apathy, paranoia, suicidality, headache, motor signs, a progressive clinical course, or a delayed onset of symptoms (e.g., after retirement from sport)].

A major gap in the literature is that there are very few published studies relating to the specificity of the proposed research criteria for TES (41, 42). This is the first study to examine the research criteria for TES (39) in men from the US general population who have intermittent explosive disorder (IED). We chose to study these men from the general population because the “behavioral” subtype of TES is defined as follows: “Being described as emotionally explosive (e.g., having a “short fuse” or being “out of control”), physically violent, and/or verbally violent, as reported by self or informant, by history of treatment, or by clinician's report. A formal diagnosis of IED would meet this criterion but is not necessary (39). We hypothesized that a substantial percentage of these men from the general population would meet the proposed research criteria for TES.

## Methods

### Participants

The National Comorbidity Survey Replication (NCS-R), conducted between February 2001 and April 2003 (43, 44), examined the prevalence and correlates of mental disorders in the United States (45–49). The interview for this survey was conducted in the homes of a nationally representative sample of adult respondents (*N* = 9,282, 4,139 men and 5,143 women) (44). The clinical sample of interest was obtained by applying a filter to the publicly available NCS-R database selecting all “male” participants meeting the criteria for a *Diagnostic and Statistical Manual of Mental Disorders, Fourth Edition* (*DSM-IV*) IED in the past year (i.e., variable = “D_IED12”). This filter resulted in the inclusion of 206 men; an incidence rate of 5.0% of all men in the database. The mean age of this sample was 32.9 years (median = 30.5, SD = 12.1, interquartile range = 23–41, range = 18–83). Their race was reported as follows: white = 65.5%, African Americans = 12.6%, Hispanic = 11.2%, Asian = 2.4%, and all other races = 8.3%. Their level of education was as follows: 0–11 years = 22.3%, 12 years = 33.0%, 13–15 years = 30.6%, and 16 or more years = 14.1%. Their employment status was as follows: employed = 73.8%, unemployed = 1.9%, and not in the labor force = 23.8%. Their relationship status was described as 56.3% married, 32.0% as never married, and 11.7% as divorced, separated, or widowed. The remaining 3,933 men were considered to be a sample representing the general population. The mean age of this sample was 44.4 years (median = 43.0, SD = 17.0, interquartile range = 31–56, range = 18–93). Their race was reported as follows: white = 74.8%, African Americans = 10.8%, Hispanic = 9.4%, Asian = 2.0%, and all other races = 3.0%. Their level of education was as follows: 0–11 years = 15.4%, 12 years = 28.9%, 13–15 years = 27.9%, and 16 or more years = 27.8%. Their employment status was as follows: employed = 72.1%, unemployed = 6.8%, not in the labor force = 20.5%, and missing = 0.5%. Their relationship status was described as 62.4% married, 22.2% never married, and 15.4% divorced, separated, or widowed.

### The NCS-R Protocol

Researchers from the Survey Research Center of the Institute for Social Research at the University of Michigan conducted the survey using laptop computer-assisted personal interviews. The core diagnostic assessment, conducted with 9,282 respondents, included the following modules: household listing, screening, depression, mania, irritable depression, panic disorder, specific phobia, social phobia, agoraphobia, generalized anxiety disorder, IED, suicidality, services, and pharmacoepidemiology. The diagnoses were derived from the World Mental Health Survey Initiative Version of the World Health Organization Composite International Diagnostic Interview, a fully structured lay-administered diagnostic interview that generates both *International Classification of Diseases, 10th Revision* (50) and *DSM-IV* (51) diagnoses. The NCS-R database is publicly available, and we accessed it at http://www.icpsr.umich.edu/icpsrweb/ICPSR/studies/20240.

### Research Criteria for Traumatic Encephalopathy Syndrome

The research criteria for “traumatic encephalopathy syndrome” (39) include three proposed core features of CTE: (i) “cognitive,” (ii) “behavioral” (i.e., anger dyscontrol), and (iii) “mood” (i.e., depression or hopelessness). We selected a sample of men who would definitively meet the core criterion for “behavioral” in that they were diagnosed with *DSM-IV* IED within the past 12 months. The “supportive features” for a diagnosis of the syndrome include impulsivity, anxiety, apathy, paranoia, suicidality, headache, motor signs, a documented decline in functioning or a progression of symptoms, or a delayed onset—such as having problems at least 2 years after the end of a career in contact sports. Two or more supportive features must be present. For the present study, we selected five of the nine supportive features, available in the NCS-R database, for the primary analyses (i.e., impulsivity, anxiety, apathy, suicidality, and headache). Other supportive features, such as paranoia, motor signs, decline in functioning, and delayed onset of symptoms were deemed to be less reliable or missing variables in the NCS-R database. The neurotrauma exposure criterion for TES is broad and diverse and includes any one of the following: (i) four of more concussions; (ii) two or more moderate or severe TBIs; (iii) involvement in “high exposure” contact sports (e.g., American football, ice hockey, lacrosse, rugby, wrestling, and soccer) for a minimum of 6 years, including at least 2 years at the college level (or higher); (iv) military service (including, but not limited to, combat exposure to blast and other explosions as well as non-combat exposure to explosives, or to combatant training or breaching training); or (v) history of any other significant exposure to repetitive hits to the head (including, but not limited to, domestic abuse, head banging, and vocational activities such as door breaching by police). This criterion could not be applied because the information was not available in the NCS-R database.

The rates of screening positively for TES are presented in two ways. First, the rate at which the men meet two of the five supportive criteria is presented. Second, the rate at which the men meet one of the five supportive criteria is presented because this simulates the normal clinical situation in which the delayed-onset criterion and/or the decline in functioning criterion would be met. The “delayed onset” criterion requires “delayed onset of clinical features after significant head impact exposure, usually at least 2 years and in many cases several years after the period of maximal exposure,” and the “documented decline” criterion requires a “progressive decline in function and/or a progression in symptoms” (39). Any former athlete or military veteran who met the neurotrauma exposure criteria and retired in their 20s or 30s, for example, who had mental health or neurological problems consistent with “TES” anytime between the ages of 40 years and the time of their death would meet the delayed-onset criterion.

## Results

The prevalence of IED in men in the National Comorbidity Survey Replication was 9.7% for lifetime, 5.0% for past year, and 2.1% for past 30 days. Lifetime diagnoses of major mental disorders, and mental disorders experienced over the past year and past 30 days, stratified by group, are presented in Table 1. Lifetime experiences with headaches, suicidality, anger, and chronic pain, stratified by group, are presented in Table 2. Compared to men in the general population, those with IED have a greater lifetime history of many disorders, including but not limited to attention-deficit/hyperactivity disorder [20.9 vs. 3.6%; χ^2^ = 135.5, *p* < 0.001, relative risk (RR) = 5.7, 95% confidence interval (CI) = 4.1–7.9], conduct disorder and oppositional defiant disorder (both 22.3 vs. 4.5%; χ^2^ = 122.1, *p* < 0.001, RR = 5.0, 95% CI = 3.6–6.7), alcohol abuse (45.6 vs. 14.5%; χ^2^ = 141.3, *p* < 0.001, RR = 3.2, 95% CI = 2.6–3.7), drug abuse (30.1 vs. 8.8%; χ^2^ = 100.4, *p* < 0.001, RR = 3.4, 95% CI = 2.7–4.3), major depressive episode (35.9 vs. 13.8%; χ^2^ = 75.5, *p* < 0.001, RR = 2.6, 95% CI = 2.1–3.2), mania (16.0 vs. 2.5%; χ^2^ = 115.6, *p* < 0.001, RR = 6.4, 95% CI = 4.3–9.3), generalized anxiety disorder (18.0 vs. 4.7%; χ^2^ = 72.2, *p* < 0.001, RR = 4.1, 95% CI = 2.9–5.7), panic disorder (12.6 vs. 2.8%; χ^2^ = 68.3, *p* < 0.001, RR = 3.8, 95% CI = 2.7–5.3), adult separation anxiety disorder (17.5 vs. 4.3%), social phobia (i.e., social anxiety disorder, 21.8 vs. 10.3%; χ^2^ = 26.9, *p* < 0.001, RR = 2.1, 95% CI = 1.6–2.8), and a specific phobia (21.4 vs. 8.3%; χ^2^ = 40.6, *p* < 0.001, RR = 2.6, 95% CI = 1.9–3.4). Over the past 12 months, those with IED were 6.9 times more likely to meet criteria for *DSM*–*IV* alcohol abuse disorder (17.5 vs. 2.5%; χ^2^ = 137.4, *p* < 0.001, RR = 6.9, 95% CI = 4.7–9.9), and 4.4 times more likely to meet criteria for *DSM*–*IV* major depressive episode (23.8 vs. 5.4%; χ^2^ = 111.4, *p* < 0.001, RR = 4.4, 95% CI = 3.3–5.8) than men from the general population.

**Table 1:** DSM-IV disorders.

	**Men with intermittent explosive disorder (*****n*** **=** **206)**			**Men from the general population (*****n*** **=** **3,933)**		
	**Lifetime**	**Past 12 months**	**Past 30 days**	**Lifetime**	**Past 12 months**	**Past 30 days**
	***n* (%)**	***n* (%)**	***n* (%)**	***n* (%)**	***n* (%)**	***n* (%)**
Substance Use Disorders						
Alcohol abuse	94 (45.6%)	36 (17.5%)	11 (5.3%)	569 (14.5%)	100 (2.5%)	31 (0.8%)
Alcohol dependence	48 (23.3%)	17 (8.3%)	7 (3.4%)	227 (5.8%)	44 (1.1%)	18 (0.5%)
Drug abuse	62 (30.1%)	16 (7.8%)	6 (2.9%)	345 (8.8%)	55 (1.4%)	20 (0.5%)
Drug dependence	28 (13.6%)	6 (2.9%)	2 (1.0%)	109 (2.8%)	15 (0.4%)	7 (0.2%)
Nicotine dependence	41 (19.9%)	26 (12.6%)	18 (8.7%)	259 (6.6%)	122 (3.1%)	79 (2.0%)
Intermittent explosive disorder	206 (100%)	206 (100%)	88 (42.7%)	197 (5.0%)	0 (0.0%)	0 (0.0%)
Mood Disorders						
Dysthymia	17 (8.3%)	12 (5.8%)	8 (3.9%)	106 (2.7%)	57 (1.4%)	26 (0.7%)
Major depressive episode	74 (35.9%)	49 (23.8%)	24 (11.7%)	543 (13.8%)	213 (5.4%)	77 (2.0%)
Bipolar I	13 (6.3%)	11 (5.3%)	7 (3.4%)	25 (0.6%)	16 (0.4%)	9 (0.2%)
Bipolar II	5 (2.4%)	5 (2.4%)	3 (1.5%)	31 (0.8%)	23 (0.6%)	13 (0.3%)
Bipolar subthreshold	20 (9.7%)	14 (6.8%)	7 (3.4%)	82 (2.1%)	42 (1.1%)	14 (0.4%)
Hypomania	5 (2.4%)	2 (1.0%)	1 (0.5%)	39 (1.0%)	16 (0.4%)	6 (0.2%)
Mania	33 (16.0%)	27 (13.1%)	12 (5.8%)	99 (2.5%)	52 (1.3%)	21 (0.5%)
Anxiety Disorders						
Generalized anxiety disorder	37 (18.0%)	26 (12.6%)	17 (8.3%)	184 (4.7%)	90 (2.3%)	39 (1.0%)
Panic attack	103 (50.0%)	43 (20.9%)	17 (8.3%)	867 (22.0%)	260 (6.6%)	82 (2.1%)
Panic disorder	26 (12.6%)	17 (8.3%)	8 (3.9%)	109 (2.8%)	50 (1.3%)	24 (0.6%)
Agoraphobia without panic disorder	11 (5.3%)	10 (4.9%)	4 (1.9%)	63 (1.6%)	37 (0.9%)	20 (0.5%)
Agoraphobia with panic disorder	7 (3.4%)	6 (2.9%)	0 (0.0%)	36 (0.9%)	22 (0.6%)	12 (0.3%)
Posttraumatic stress disorder	17 (8.3%)	10 (4.9%)	2 (1.0%)	119 (3.0%)	58 (1.5%)	35 (0.9%)
Adult separation anxiety disorder	36 (17.5%)	11 (5.3%)	8 (3.9%)	169 (4.3%)	47 (1.2%)	18 (0.5%)
Social phobia	45 (21.8%)	31 (15.0%)	14 (6.8%)	405 (10.3%)	208 (5.3%)	89 (2.3%)
Specific phobia	44 (21.4%)	31 (15.0%)	22 (10.7%)	328 (8.3%)	197 (5.0%)	137 (3.5%)
Adolescent/Developmental Disorders						
Attention deficit disorder	43 (20.9%)	26 (12.6%)	NA	143 (3.6%)	61 (1.6%)	NA
Conduct disorder	46 (22.3%)	8 (3.9%)	NA	177 (4.5%)	14 (0.4%)	NA
Oppositional defiant disorder	46 (22.3%)	5 (2.4%)	NA	177 (4.5%)	17 (0.4%)	NA

**Table 2:** Headaches, chronic pain, anger, and suicidal behavior.

	**Men with intermittent**				**Men from the general**			
	**explosive disorder (*****n*** **=** **206)**				**population (*****n*** **=** **3,933)**			
	**Endorsed**		**Missing**		**Endorsed**		**Missing**	
**Condition**	***f***	**%**	***f***	**%**	***f***	**%**	***f***	**%**
Headaches								
Ever had frequent or severe headaches	54	26.2	14	6.8	396	10.1	1,728	43.9
Still have severe headache or received treatment	31	15.0	152	73.8	206	5.2	3,537	89.9
Chronic pain								
Ever had chronic back/neck problems	66	32.0	14	6.8	707	18.0	1,728	43.9
Still have back/neck problems or receive treatment in past year	44	21.4	140	68.0	474	12.1	3,226	82.0
Ever had any other chronic pain	26	12.6	14	6.8	272	6.9	1,728	43.9
Still have chronic pain or received treatment in past year	19	9.2	180	87.4	176	4.5	3,661	93.1
Ever had arthritis/rheumatism	40	19.4	14	6.8	533	13.6	1,732	44.0
Anger Attacks (Ever in Life….)								
Anger attack leading to breaking item of some value	181	87.9	0	0	1,074	27.3	3	0.1
Anger attack leading to hitting/attempt hitting person	137	66.5	0	0	977	24.8	5	0.1
Anger attack leading to threat of harm to person	43	20.9	137	66.5	599	15.2	979	24.9
Irritability and anger in past month								
Feel irritable/grumpy^*^	104	50.5	9	4.4	595	15.1	1,235	31.4
Feel mad/angry	95	46.1	9	4.4	398	10.1	1,234	31.4
Feel angry and out of control	33	16.0	27	13.1	64	1.6	2,318	58.9
Feel urge hit/push/hurt someone	26	12.6	27	13.1	58	1.5	2,318	58.9
Feel urge to break/smash something	32	15.5	27	13.1	61	1.6	2,318	58.9
Suicidal Ideation and Behavior								
Ever seriously thought about committing suicide	57	27.7	36	17.5	395	10.0	697	17.7
Seriously thought about committing suicide in past 12 months	11	5.3	148	71.8	58	1.5	3,538	90
Ever made a plan for committing suicide	27	13.1	148	71.8	121	3.1	3,538	90
Made a suicide plan in the past 12 months	4	1.9	179	86.9	14	0.4	3,812	96.9
Ever attempted suicide	20	9.7	148	71.8	97	2.5	3,539	90
Attempted suicide in the past 12 months	3	1.5	186	90.3	11	0.3	3,836	97.5

%, percentage; f, frequency; n, number.

* *Those who rated experiencing irritability or anger as some of the time, most of the time, or all of the time were included; GAD, Generalized Anxiety Disorder; IED, intermittent Explosive Disorder. Missing values included values missing from the system, don't know responses, and/or refused to respond responses*.

The percentages of men with IED meeting supportive criteria for TES are presented in Table 3. “Impulsivity,” as reflected by a diagnosis of alcohol abuse or drug abuse in the past year, was present in 20.4%. Problems with anxiety were present in 49.0%, and suicidality was present in 5.3%. Apathy was reported by 10.7%. A significant problem with headaches was reported by 15.0%. Overall, 27.3% met the criteria for two or more supportive features for the syndrome and thus when combined with their diagnosis of IED would meet the clinical criteria for TES. We could not apply the criteria relating to delayed onset (e.g., anger control problems in a middle-aged man who played college football) or progressive worsening of symptoms (i.e., over at least a 1-year duration). Assuming that one of those two criteria was met, then only one additional criterion from Table 3 would be necessary to meet research criteria for the syndrome. The proportion of the IED sample who met one or more of the criteria was 65.0%.

**Table 3:** Percentage of men meeting research criteria for supportive features of traumatic encephalopathy syndrome.

	**Intermittent explosive disorder (*n* = 206)**	**General population (*n* = 3,933)**
“Impulsivity. Impaired impulse control, as demonstrated by new behaviors, such as excessive gambling, increased or unusual sexual activity, substance abuse, excessive shopping or unusual purchases, or similar activities (39).” Definition: DSM-IV diagnosis of alcohol abuse in past year (D_ALA12) or drug abuse in past year (D_DRA12).	20.4%	3.4%
“Anxiety. History of anxious mood, agitation, excessive fears, or obsessive or compulsive behavior (or both), as reported by self or informant, history of treatment, or clinician's report. A formal diagnosis of anxiety disorder would meet this criterion but is not necessary (39).” Definition: DSM-IV diagnosis, in the past year, of generalized anxiety disorder (D_GAD12), agoraphobia without panic disorder (D_AGO12), agoraphobia with panic disorder (D_AGP12), panic disorder (D_PDS12), social phobia (i.e., social anxiety disorder; D_SO12), posttraumatic stress disorder (D_PTS12), or rating any of the following as 1 = often in the past month: nervousness, fidgety, tense (SC9D); worry too much about things (NSD1F); suddenly scared no reason (NSD1B); or feel frightened (NSD1H).	49.0%	14.9%
“Apathy. Loss of interest in usual activities, loss of motivation and emotions, and/or reduction of voluntary, goal-directed behaviors, as reported by self or informant, history of treatment, or clinician's report (39).” Definition: No interest in things over the past month (rated as 1 = often; NSD1G).	10.7%	2.2%
“Suicidality. History of suicidal thoughts or attempts, as reported by self or informant, history of treatment, or clinician's report (39).” Definition: Seriously thought about committing suicide in past 12 months (SD3), made a suicide plan in past 12 months (SD5), or attempted suicide in past 12 months (SD10).	5.3%	1.5%
“Headache. Significant and chronic headache with at least one episode per month for a minimum of 6 months (39).” Definition: Reports a current problem with severe headaches and/or treatment for headaches (CC4C).	15.0%	5.2%
One or more of the above criteria are met.	65.0%	20.1%
Two or more of the above criteria are met.	27.3%	5.7%

*Variable names from the publicly available NCS-R database are provided*.

## Discussion

This is the first study to examine a proposed set of research criteria for the diagnosis of TES (39) in a sample of men from the general population with IED. There were three primary important findings. First, anger attacks are common in men in the general population, with one in four men reporting them at some point during their lifetime (Table 2). Second, in the general population, the lifetime prevalence of IED is 9.7% in the present study, and men with this disorder have a high lifetime prevalence of other disorders that have been proposed to be clinical features of CTE and TES, such as alcohol abuse (45.6%), drug abuse (30.1%), and major depressive episode (35.9%). In other words, men in the US general population with severe anger control problems are likely to experience other symptoms, problems, and disorders that researchers have proposed to be characteristic of CTE and TES (Tables 1, 2). Finally, the rate of meeting the symptom criteria for TES in men from the US general population who have serious anger control problems, during the past year, is high (Table 3). The average age of this sample was 33, 50% were between the ages of 23 and 41 years, and 25% were older than 41 years. In other words, a large percentage of this sample was of a similar age of men who have retired from contact or collision sports or retired from the military. As such, if we assume the “delayed onset” supportive feature is met, then only one additional supportive feature is necessary to diagnose TES. As such, approximately two of three men from the general population, who have serious anger control problems, meet the proposed research diagnostic criteria for TES.

It is essential to appreciate that anger dyscontrol and aggressive behavior are complex and multifactorial in causation. There are many reasons why former athletes or military veterans might have anger control problems. Temperamental (52–54) and personality (55, 56) factors have been linked to risk of anger dyscontrol and aggression. Adverse events in childhood, such as abuse and neglect, have been associated with increased risk of future anger control problems (57–59). Men who had abusive or aggressive fathers are statistically more likely to also be abusive or aggressive (57). Some boys might choose certain high contact, collision, or combat sports in part due to innate aggressiveness (60, 61). As such, a certain degree of anger dyscontrol and aggressiveness may represent longstanding behavioral and personality characteristics in some former athletes, as has been speculated by authors writing about former boxers (2, 4, 5, 8, 62–64). These longstanding characteristics could be amplified or exacerbated by life stress, depression, anxiety, substance abuse, chronic cumulative brain damage, and a number of neurological and neurodegenerative diseases. Life stress (65), financial problems (66), marital problems (66), and substance abuse (67, 68) are all associated with anger control problems. Military veterans with posttraumatic stress disorder frequently have anger control problems (69–71). Men who develop a depressive disorder are also at risk of having anger attacks (72, 73). Anger attacks in men with depression have been assumed to be related to the depressive disorder, as opposed to reflecting a primary underlying IED. People with TBIs sometimes develop problems with anger dyscontrol and aggressiveness, particularly after sustaining a single severe TBI (74, 75). However, the associations between cumulative mild injuries to the brain and anger or aggression are not well-understood. Finally, problems with anger and aggression can occur as a result of a neurological disorder, such as a stroke (76–78), or during the course of a neurodegenerative disease, such as Alzheimer disease (79, 80).

Modern researchers studying CTE have emphasized psychiatric and behavioral problems as being common (39), and those who are younger and who have less neuropathology have been conceptualized as being more likely to have this proposed “mood” or “behavioral” subtype or phenotype of TES and CTE (81). The mechanisms by which small amounts of p-tau in specific brain regions drive complex changes in behavior, such as depression and anger control problems, have not been studied in a meaningful way and are unknown. Nonetheless, modern researchers seem to suggest that virtually any clinical or psychosocial problem present prior to death in someone who has CTE neuropathology in their brain identified on postmortem examination must have that problem as a direct result of the CTE neuropathology. For example, the two leading research groups in the United States have asserted that clinical features of CTE include (i) depression and anxiety (18, 82, 83); (ii) suicidality (18, 81, 83–87); (iii) poor financial decisions, financial problems, and bankruptcy (82); (iv) gambling (39); (v) excessive shopping or unusual purchases (39); (vi) marital problems, separation, and divorce (87); and (vii) substance abuse (39). This approach to defining clinical features represents association by assertion, or *circulus in probando*, as opposed to being based on empirical research or rigorous clinicopathological correlation.

In their review of all known cases of CTE, published in 2009, McKee et al. (20) documented that 17 of the 41 cases (41.5%) published in the twentieth century had a personal history of aggression or violence. We reexamined the 41 case studies, and without question, some former boxers believed to have CTE had documented anger control problems and violent behavior during their lifetime (4–6, 8, 10, 64). In contrast, other former boxers were described as showing euphoria (6, 15), a child-like demeanor (4), or “fatuous cheerfulness” (6). The demographic and clinical characteristics of the 17 cases with aggressive behavior are summarized in Table 4. According to the new proposed criteria for TES, a former boxer (or contact sport athlete) could be diagnosed as having the behavioral variant of TES if he had developed clinical problems “at least 2 years after a period of maximal exposure” (39) and had any one of the following problems: excessive gambling, unusual sexual activity, excessive shopping or unusual purchases, anxiety, excessive fears, obsessive-compulsive disorder, any anxiety disorder, or suicidality. To our knowledge, there was never a case that matched any of those characteristics in the twentieth century, based on our review of the case information presented in the tables in McKee et al. and our review of the six published studies that reported the case histories of those 17 people. As seen in Table 4, the aggressive behavior and volatility displayed by these former boxers generally co-occurred with other obvious neurological signs of brain damage, such as dysarthric speech, gait problems, and parkinsonism, and virtually all had cognitive impairment or dementia (2, 4, 5, 10, 64). Moreover, authors sometimes noted that aggressive behavior seemed to be a longstanding problem (4, 5, 10), perhaps contributing to their chosen career of boxing.

**Table 4:** Twentieth century case studies of presumed CTE with a history of violence or aggressiveness (*n* = 17) from McKee et al. (20).

**Case** **#**	Year^*^	**Age** **sport** **began**	**Years in sport**	**Initial** **symptoms** **and problems**	**Age of onset** **of symptoms**	**Years between retirement and symptoms**	**Interval between symptom onset and death**	**Age at death**	**Cognitive changes**	**Memory loss**	**Dementia**	**Parkinsonism or** **obvious neurological** abnormality^***^	**CTE pathology (88)**
8	1967	16	7	Cognitive decline, hemiparesis	25	1	33	58	Yes	Yes	Yes	Yes	N/A
10	1968	15	20	Headaches	36	0	10	46	No	No	No	Yes	N/A
11	1968	19	12	Headaches	31	0	15	46	Yes	Yes	No	Yes	N/A
12	1968	16	16	Slurred speech, gait change	40	8	5	45	Yes	Yes	No	Yes	N/A
15	1973	11	14	Violent outbursts	25	0	38	63	Yes	Yes	No	Yes	Yes CTE
17	1973	16	14	Confusion, falls	30	0	33	63^§^	Yes	Yes	No	Yes	Yes CTE
19	1973	18	18	Irritability, memory loss, aggression	36	0	25	61	Yes	Yes	No	Yes	No ARTAG
20	1973	13	25	Gait, speech	37	0	46	83	Yes	Yes	No	Yes	Yes CTE
21	1973	16	20	Dysphoria, violence	54	18	8	62	Yes	Yes	Yes	Yes	Yes CTE
22	1973	17	23	Ataxia, falls, weakness	60	20	11	71	Yes	No	No	Yes	Yes CTE
24	1973	NR	NR	Memory loss	40	NR	27	67	Yes	Yes	Yes	NR	Yes CTE/AD
25	1973	NR	NR	Confusion	48	NR	19	67	Yes	Yes	Yes	Yes	No ARTAG
26	1973	14	16	Speech, delirium	43	4	14	57^§^	Yes	Yes	Yes	Yes	No AD
28^**^	1973	NR	NR	Aggression	NR	NR	NR	91	No	No	No	No	No Diagnosis
32	1992	NR	>25	NR	NR	NR	NR	63	Yes	Yes		Yes	N/A
34	1996	NR	15	NR	NR	NR	NR	33	Yes	Yes	Yes	NR	N/A
41	1999	NR	10	Cognitive decline, ALS-like syndrome	64	NR	NR	67	Yes	Yes	Yes	Yes	N/A

This information was derived from Table 2 in McKee et al. (20). All cases were reported to have been boxers with the exception of a circus clown who was an achondroplastic dwarf, aged 33 at the time of death, who had 15 years of exposure as a circus clown and participation in dwarf throwing competitions reported by Williams and Tannenberg (89).

* 1967 = Constantinidis and Tissot (90), 1968 = Payne (64), 1973 = Corsellis et al. (4), 1992 = Hof et al. (91), 1996 = Williams and Tannenberg (89), 1999 = No listed authors (92), 2018 = Goldfinger et al. (88);

** Case 28 said to have boxed professionally in his youth. He was described as being “considered active and mentally alert up to the time of his death” (4); AD, Alzheimer's disease; ARTAG, Aging-related tau astrogliopathy; CTE, chronic traumatic encephalopathy; N/A, not applicable; NR, not reported;

§ Case 17 “Age at Death” reported as 63 years in Corsellis et al. (4) and 62 years in Goldfinger et al. (88); Case 26 “Age at Death” reported as 57 years in Corsellis et al. (4) and 56 years in Goldfinger et al. (88);

*** *Obvious neurological abnormality includes two or more of the following: movement abnormalities, decreased facial movement, slowed movements, tremor, rigidity, falls, ocular abnormalities, ptosis, reduced upgaze, gait problems: staggered gait, slowed gait, shuffled gait, ataxia, reduced coordination, speech changes: slowed speech, slurred speech, dysarthria, dysphagia, spasticity*.

Our study has three important limitations. First, we have no information on the subjects' concussion history, and it is likely that some of the men in our case series experienced one or more concussions during the course of their lives because concussions are very common in men in the general population (93, 94). Second, we were unable to study the “exposure history” criterion in the research definition of TES. It is possible that some of the men included in this study would have met the exposure criterion. It is important to appreciate that *all* former professional soccer players, hockey players, boxers, and American or Canadian football players meet the exposure criteria for repetitive neurotrauma. The exposure criterion is very inclusive and simply requires that the person played one or more sports (e.g., boxing, American football, ice hockey, lacrosse, rugby, wrestling, or soccer), for a minimum of 6 years [with 2 at the college level (or equivalent) or higher], which resulted in “multiple impacts to the head” that can be concussions or “subconcussive trauma” (i.e., with no clinical symptoms) (39). Moreover, military service or police training involving exposure to blasts, explosives, combat, or breaching is listed as a source of exposure sufficient to meet criteria. It is very likely that some men who participated in the NCS-R study played contact sports, at least at the high school level. No information relating to lifetime history of sports participation was available in the NCS-R database. Finally, we were not able to align precisely the results of the NCS-R interviews on to the research criteria for TES, which are broader and more inclusive than what we could study. We did not include two categories of supportive features: (i) paranoia and (ii) motor signs. Moreover, within the category of impulsivity, we did not include “excessive gambling,” “increased or unusual sexual activity,” or “excessive shopping or unusual purchases.” If we had more variables that aligned with all the supportive features criteria, the rate of identifying TES in this sample would have been greater.

In conclusion, we examined the proposed research criteria for the behavioral subtype of TES in a large sample of men with serious anger control problems who were selected from a nationally representative sample of men from the US general population who underwent a thorough in-person psychiatric interview yielding *DSM-IV* diagnoses. Using liberal criteria, we discovered that approximately two of three of these men could be identified as having TES. Researchers have not established a clinicopathological correlation between anger control problems and the region-specific accumulation of hyperphosphorylated tau believed to characterize CTE (19), so researchers and clinicians should not assume that anger control problems in a former athlete or military veteran are caused by CTE neuropathology. Anger control problems described in the case histories of boxers in the twentieth century were not considered to be a “behavioral” phenotype or subtype of CTE, nor were they described as a core clinical feature. More research is needed to examine risks for misdiagnosing TES, which appear considerable based on the results of this study. In addition, more research is needed to determine whether anger dyscontrol is a clinical phenotype of CTE.

## Data Availability Statement

Publicly available datasets were analyzed in this study. This data can be found here: http://www.icpsr.umich.edu/icpsrweb/ICPSR/studies/20240.

## Ethics Statement

The studies involving human participants were reviewed and approved by University of Michigan. The patients/participants provided their written informed consent to participate in this study.

## Author Contributions

GI and AG contributed to the conception and design of the study. GI analyzed the database. All authors drafted and revised the manuscript and approved the submitted version.

## Conflict of Interest

GI serves as a scientific advisor for BioDirection, Inc., Sway Operations, LLC, and Highmark, Inc. He has a clinical and consulting practice in forensic neuropsychology, including expert testimony, involving individuals who have sustained mild TBIs (including athletes). He has received research funding from several test publishing companies, including ImPACT Applications, Inc., CNS Vital Signs, and Psychological Assessment Resources (PAR, Inc.). He has received research funding as a principal investigator from the National Football League, and salary support as a collaborator from the Harvard Integrated Program to Protect and Improve the Health of National Football League Players Association Members. He acknowledges unrestricted philanthropic support from ImPACT Applications, Inc., the Heinz Family Foundation, and the Mooney-Reed Charitable Foundation. AG has a clinical practice in neuropsychology involving individuals who have sustained sport-related concussion (including current and former athletes). He has been a contracted concussion consultant to Rugby Australia since July 2016. He has received travel funding from the Australian Football League (AFL) to present at the Concussion in Football Conference in 2013 and 2017. Previous grant funding includes the NSW Sporting Injuries Committee, the Brain Foundation (Australia), and the Hunter Medical Research Institute (HMRI), supported by Jennie Thomas, and the HMRI, supported by Anne Greaves. He is currently funded through an NHMRC Early Career Fellowship, and Hunter New England Local Health District, Research, Innovation and Partnerships Health Research & Translation Center and Clinical Research Fellowship Scheme, an Australian-American Fulbright Commission Postdoctoral Award, and the University of Newcastle's Priority Research Center for Stroke and Brain Injury. The handling editor declared a past co-authorship with one of the authors (AG).
